# Use of benzodiazepines in psychosis and bipolar disorder by tunisian psychiatrists

**DOI:** 10.1192/j.eurpsy.2021.1297

**Published:** 2021-08-13

**Authors:** M. Lagha, U. Ouali, F. Nacef

**Affiliations:** Department Of Psychiatry A, Razi hospital, Manouba, Tunisia

**Keywords:** Benzodiazepines, psychosis, bipolar disorder

## Abstract

**Introduction:**

Benzodiazepines (BZD) are psychotropic drugs prescribed in psychiatry for their anxiolytic, hypnotic and sedative properties. Since anxiety, agitation and insomnia are common in psychoses and mood disorders, BZDs are frequently prescribed in the treatment of these pathologies. Guidelines remain rare with regard to the use of BZDs in the treatment of psychosis and bipolar disorder.

**Objectives:**

Our study aimed to evaluate BZDs prescribing practices in psychoses and bipolar disorder and to assess the specific risks related to the use of these molecules in the population suffering from severe mental disorder.

**Methods:**

This is a descriptive cross-sectional study conducted through a Google-forms self-administered questionnaire, intended for psychiatrists and psychiatric residents, over a period of two months, from April 1 to May 31, 2019.

**Results:**

One hundred physicians practicing in psychiatry answered our questionnaire. The response rate was 28%. BZDs were prescribed during thymic or psychotic relapses by 88.6% of the participants. During relapses, the main indication for BZDs was anxiety (81.3%), insomnia (80.2%), and catatonia (59.4%). Among the participants, 24.8% indicated that they maintained a long-term treatment with BZDs in patients with psychosis, and 11.4%in patients with bipolar disorder. The participants estimated that the long-term use of BZDs in patients with severe mental disorder represented an increased risk of : dependence (94.3%), behavioral disinhibition (30.5%), suicide (22.9%), anger, hostility and violence (31.4%).

**Conclusions:**

Few guidelines concern the use of BZDs in psychosis and bipolar disorder. However, this prescription remains very frequent in current practice, with clinical and therapeutic features specific to this population.

